# Supplementary material for: Surface Functionalization of Black Phosphorus via Amine Compounds and Its Impacts on the Flame Retardancy and Thermal Decomposition Behaviors of Epoxy Resin
Source: Polymers (Basel). 2021 Oct 21;13(21):3635. doi: 10.3390/polym13213635 (PMC8588435; doi:10.3390/polym13213635)
Supplement: Supplementary file 1 [file polymers-13-03635-s001.zip › polymers-1403014-supplementary.pdf]

Supporting Information

Surface functionalization of black phosphorus via amine compounds and its impacts on the flame retardancy and thermal decomposition behaviors of epoxy resin

Shaoling Lin[a,b], Boqing Tao[b], Xiaomin Zhao[b]*, Guohua Chen[b], De-Yi Wang[c]*

a College of Chemical Engineering, Huaqiao University, Xiamen 361021, China

b College of Materials Science and Engineering, Huaqiao University, Xiamen 361021, China

c IMDEA Materials Institute, C/Eric Kandel, 2, 28906 Getafe, Madrid, Spain

*Corresponding author.

E-mail addresses: xiaominelena@163.com (Xiaomin Zhao), deyi.wang@imdea.org (De-Yi Wang).

Table S1. Elemental distribution of BP and BP-NH_2 nanosheets detected by XPS analysis.

Sample	Element content (%)			
	C	O	N	P
**BP**	9.83	11.06	1.46	77.65
**BP-DETA**	42.04	29.9	14.81	13.25
**BP-PPDA**	44.08	27.05	6.07	22.79
**BP-Pid**	41.96	21.82	8.18	28.04

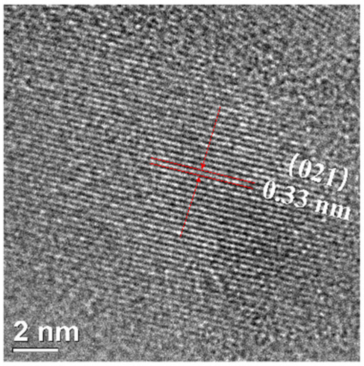

Figure 1. The high-resolution TEM images of BP.

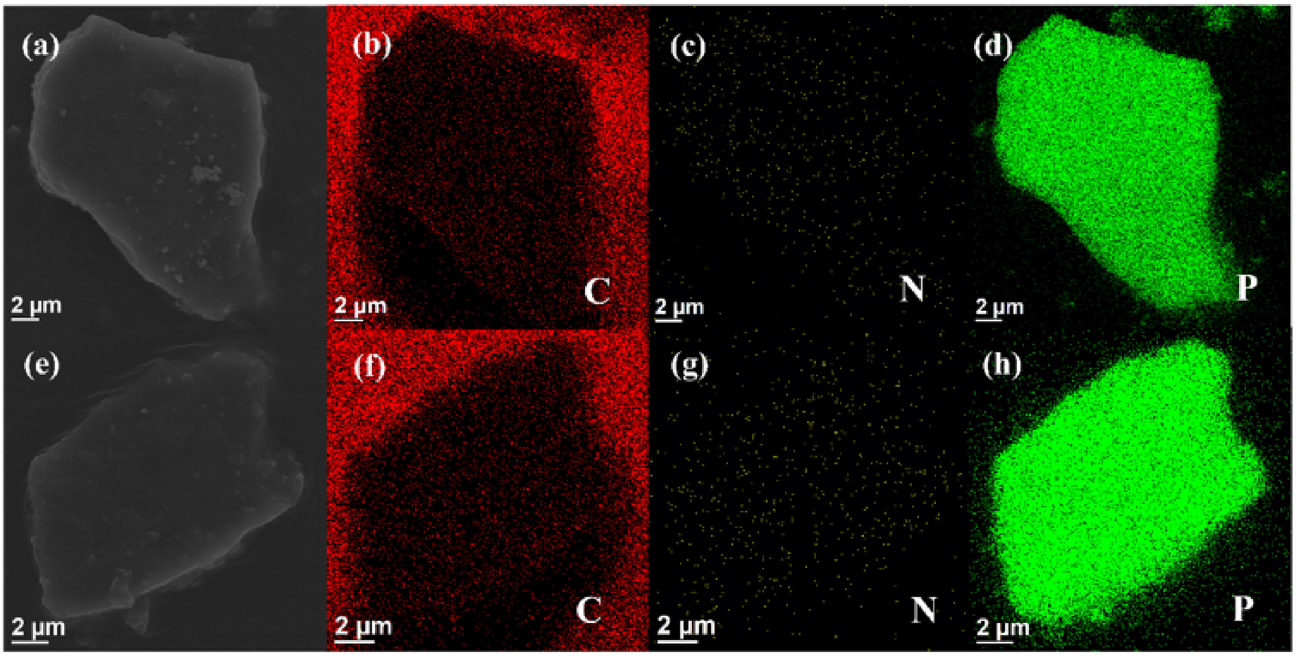

Figure S2. (a) SEM image of BP-PPDA nanosheets and the corresponding elemental mapping images of (b) carbon (C), (c) nitrogen (N), (d) phosphorus (P); (e) SEM image of BP-Pid nanosheets and the corresponding elemental mapping images of (f) carbon (C), (g) nitrogen (N), (h) phosphorus (P).
